# Micronuclei: origins, assays, mechanisms, diseases and treatments

**DOI:** 10.1038/s41392-025-02538-8

**Published:** 2026-03-26

**Authors:** Hailong Duan, Xin Peng, Sha Qin, Yanmin Zhou, Desheng Xiao, Yongguang Tao, Shuang Liu

**Affiliations:** 1 The Affiliated Cancer Hospital of Xiangya School of Medicine, Central South University/Hunan Cancer Hospital, Hunan Key Laboratory of Cancer Metabolism Changsha, Changsha, Hunan China; 2 Department of Pathology, Xiangya Hospital, Central South University, Changsha, Hunan China; 3 Key Laboratory of Carcinogenesis and Cancer Invasion of Ministry of Education, Cancer Research Institute, Central South University, Changsha, Hunan China; 4 Department of Oncology, Xiangya Hospital, Central South University, Changsha, China; 5 Department of Pathology, School of Basic Medicine, Central South University, Changsha, Hunan China; 6 Department of Thoracic Surgery, Hunan Key Laboratory of Early Diagnosis and Precision Therapy in Lung Cancer, Second Xiangya Hospital, Central South University, Changsha, Hunan China; 7 Institute of Medical Sciences, Xiangya Hospital, Central South University, Changsha, China

**Keywords:** Genomic instability

## Abstract

Micronuclei are small, independent cytoplasmic structures containing nuclear material. They typically form during cell division due to DNA damage or division abnormalities, serve as biomarkers of genetic damage, and are closely associated with chromosomal instability (CIN). Emerging evidence suggests that micronuclei actively promote and exacerbate CIN, with significant implications in disease pathology and potential therapeutic applications. This review provides a comprehensive overview of micronuclei by exploring their origins, formation mechanisms, and functional consequences, and detailing the fate of micronuclei post-formation, which is essential for elucidating their role in genomic instability and potential therapeutic implications. Furthermore, micronuclei can contribute to extreme chromosomal shattering and genomic instability. These processes are increasingly recognized as critical contributors to disease progression, particularly in cancer. Although micronuclei have traditionally been viewed as markers of genomic instability, recent evidence suggests that they may also serve functional roles. Their potential use as treatments for certain diseases appears theoretically feasible; however, challenges remain in selectively targeting cells to induce the formation of favorable micronuclei and maintain optimal immune responses. Addressing these questions could open new avenues for therapeutic interventions.

## Introduction

Micronuclei are nucleated structures containing lagging chromosomes or chromosomal fragments enclosed by a nuclear membrane. As distinct compartments within the cytoplasm, they can be readily visualized using techniques such as Giemsa, DAPI, or Hoechst staining.^1,2^ The presence of micronuclei often indicates chromosomal instability (CIN) in cells, which refers to abnormal changes in the number or structure of chromosomes during cell division, including chromosome loss, increase, or structural rearrangement. CIN and micronuclei are important characteristics of tumor cells, while they do exist to a very low degree in normal cells. Interestingly, the prefix “micro” in micronuclei may be somewhat misleading, as these structures are typically one-sixth to one-third the size of the primary nuclei—significantly larger than what is usually implied by “micro,” which often suggests something exceedingly small, such as one percent of a standard size or smaller.^3^

Among many reasons that lead to micronuclei formation, radiation and genotoxic drugs capable of damaging chromatin DNA are the most common and direct causes.^4^ Internal cellular factors also serve as crucial stimulators of micronuclei formation, such as loss of cell cycle checkpoint function caused by epigenetic defects in the centromere or chromosome fusion events caused by telomere erosion.^5,6^ These examples show that micronuclei are one of the results or markers of CIN. Nevertheless, growing evidence shows that micronuclei not only arise as a consequence of CIN but also actively contribute to the progression of genomic instability.^7,8^ Research on micronucleus has expanded beyond mere observation, and purifying micronuclei from cells and performing assays such as proteomics have become emerging topics.^4^

This review goes over and summarizes the research history, findings, and progress of micronuclei, which we mainly focus on the formation process and fate of micronuclei, and particularly discusses the regulatory factors of micronuclei. Due to the fact that a considerable amount of research on micronuclei has been conducted in tumor cells, these contents are expected to provide valuable clues for the diagnosis and prevention of tumors. We list some of the features of micronuclei here (Table 1).

**Table 1:** Characteristics of micronucleus

Parameters	Characteristics	References
Size	1–5 μm	^3^
Shape	Any shape, mostly oval or round	^72^
Origins	Lagging chromosomes, DNA double-strand breakage, nuclear budding	^29^
Inclusion	One or several chromosomes, chromosome fragments	^281^
Location	Near the primary nucleus	^81^
Membrane	Fragile, “non-core” nuclear envelope proteins assemble defectively, functional nuclear pore complex decreased, spontaneous disruption in interphase	^20,77–79^
Fate	Reincorporation, persistence, rupture, degradation, extrusion	This text
Markers	γ-H2AX positive, cGAS positive, Centromere-specific DNA positive, H2B positive	^20,25,96^
Observation conditions	Giemsa staining, DAPI, Hoechst	^117,145,235^
Downstream pathways	cGAS-STING activation, AIM2 inflammasome activation,	^24,282^
Inheritance	Bilateral inheritance, unilateral inheritance	^283^

## The centennial history of micronuclei

The discovery of micronuclei dates back to the 1890s when William Howell identified chromatin fragments in cat erythrocytes, proposing that they were remnants of the nucleus that had been extruded and could persist in the erythrocyte until the cell died.^9^ Later, Justin Jolly observed similar nuclear fragments in phagocytes, supporting Howell’s findings. In recognition of this discovery, the erythrocyte nuclear fragments were named the “Howell-Jolly bodies” and were later classified as micronuclei.^10^ Around the same time, pathologist von Hansemann discovered what he termed a “lost chromosome”—a structure he suggested could exist independently of the nucleus.^11^ A decade later, Theodore Boveri observed that, during cell division in sea urchin eggs, certain chromosomes failed to be distributed to daughter cells and instead remained in the cytoplasm after division.^1,12^

These pioneering discoveries laid a solid foundation for modern micronuclei research, many of whose principles remain relevant today. However, research progressed slowly due to limitations in early microscopic techniques and the incomplete understanding of genetic changes associated with micronuclei. It was not until the mid-20th century that Evans and colleagues demonstrated that ionizing radiation could induce micronuclei formation in alfalfa root and whisker cells, establishing a link between micronuclei and radiation-induced chromosomal damage. Despite its significance, their research remained focused on plant cells.^13^

In the 1970s, Matter and Schmid expanded micronuclei studies to rodent bone marrow cells, using micronuclei frequency to evaluate chemical genotoxicity and laying the groundwork for the development of micronucleus assays.^14^ However, the emergence of the micronuclei assay did not immediately gain widespread attention; after all, detecting micronuclei in bone marrow cytoplasm required the killing of experimental animals, which is burdensome for researchers in terms of expense and time. The transition to using peripheral lymphocytes in micronuclei studies alleviated some of the challenges, but measuring micronuclei frequency remained problematic due to interference from cell proliferation and division. This issue was addressed with the introduction of cytochalasin B, which inhibits cytoplasmic division while allowing nuclear division. This advancement enabled Fenech and Morley to develop a reliable in vitro micronuclei assay.^15,16^ Today, micronuclei assays have been widely adopted in fields such as environmental pollution assessments, chemical and drug genotoxicity testing, and even the diagnosis of blood diseases.^17,18^ The World Health Organization (WHO) has listed micronuclei assay as one of the mandatory toxicological tests for chemicals, refining its protocols through multiple iterations.^19^

Advancements in microscopy and sequencing technologies have further enhanced the understanding of genomic disruptions caused by micronuclei. A particularly striking discovery is their involvement in chromothripsis, a catastrophic genomic event characterized by localized chromosomal rearrangements occurring in a single cellular crisis. With its term derived from Greek words for “chromosome” and “shattering into pieces”, chromothripsis is often driven by the structural fragility of the micronuclei membrane.^20,21^ Using the Look-Seq technique, Zhang et al. directly visualized chromosome-shattering rearrangements following micronuclei rupture, providing clear evidence of the disruptive effects of micronuclei-isolated chromosomes on genomic integrity.^22^ Beyond genomic instability, recent studies have established a connection between micronuclei and innate immune responses, including the activation of cGAS-STING signaling pathway.^23,24^

**Fig. 1:**
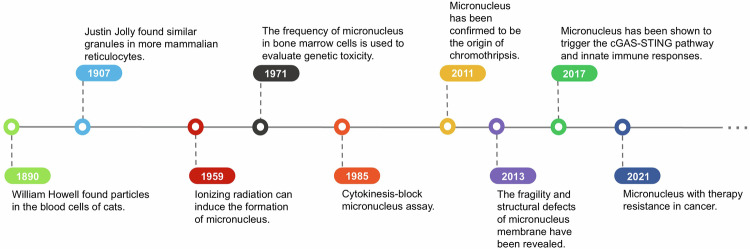
Key milestones in micronucleus research. Micronuclei were first discovered in 1890 when Howell identified particles in feline blood cells. In 1907, Jolly observed similar granules in mammalian reticulocytes. In 1959, the role of ionizing radiation in micronucleus formation was elucidated. In 1971, micronucleus frequency in bone marrow cells was used for genetic toxicity assessment, and the cytokinesis-block micronucleus assay was introduced in 1985. In 2011, micronuclei were confirmed as the origin of chromothripsis. Further research in 2013 revealed micronucleus membrane fragility and structural defects. In 2017, it was reported that micronuclei activate the cGAS-STING pathway, triggering innate immune responses. In 2021, micronuclei were associated with drug resistance in patients with cancer

In summary, the frequency of groundbreaking research on micronuclei has increased over the past decade as knowledge about micronuclei has evolved. Here, we have listed milestone events in the development of micronuclei (Fig. 1).

## Micronucleus formation

The precise mechanism underlying micronuclei formation remains unclear. Various factors contribute to their formation in human cells under different cellular conditions, and recent studies have identified several key factors that increase micronuclei frequency (Table 2). Specially, micronuclei form during cellular mitosis and are primarily associated with two major mechanisms: (1) mitotic defects leading to chromosome lagging and (2) DNA double-strand breaks (DSBs).^25,26^ Moreover, some published reviews have also proposed nuclear budding as a potential contributor to micronuclei formation. Given this perspective, nuclear budding is also considered within the scope of this discussion.^27^

**Table 2:** Newly discovered factors in recent years that can induce micronucleus formation

Type of influencing factors		Experimental population	Sample size	Experimental cell type	Reference
Dietary factors	Animal fat edible oil	Non-smokers	116	Buccal cells	^284^
	Sports supplements	Fitness personnel	147	Buccal cells	^285^
	Cigarettes	Women before and after menopause	50	Cervical cells	^286^
Chemical factors	Petroleum compounds	Schoolchildren	54	Buccal cells and peripheral blood cells	^287^
	Paroxetine	NA	NA	Lymphocytes	^288^
	Chromate	Chromate exposed workers	455	Peripheral blood cells	^289^
	TIO2	Pigment factory workers	15	Buccal cells	^290^
Environmental factors	Heavy metal (Selenium, mercury, manganese, lead, magnesium)	Residents of mining areas	306	Peripheral blood cells	^291^
	Benzene	Benzene-exposed workers	294	Peripheral blood cells	^292^
	Welding fumes	Welders	98	Peripheral blood cells	^293^
	Coal mine	Coal miners	40	Buccal cells	^294^
		Residents of coal mining areas	150	Lymphocytes and buccal cells	^295^
	Carcinogenic polycyclic aromatic hydrocarbons	Oven workers	364	Lymphocytes	^296^
	Pesticide	Horticulturists	42	Buccal cells	^297^
	Lead	Battery factory workers	1176	Peripheral blood cells	^298^
	Heavy metal (copper, iron, arsenic)	Residents of mining areas	60	Peripheral blood cells	^299^
	Marble	Marble processing factory workers	48	Lymphocytes and buccal cells	^300^
	Anesthetics	Operating room staff	193	Buccal cells and peripheral blood cells	^301^
	Tungsten-molybdenum	Children in mining areas	26	Buccal cells	^302^
	Industrial waste gas	Residents of industrial areas	26	Buccal cells	^303^
	Mobile phone radiation	Mobile phone users	41	Buccal cells	^304^

### Mitotic defects induce micronucleus formation

**Fig. 2:**
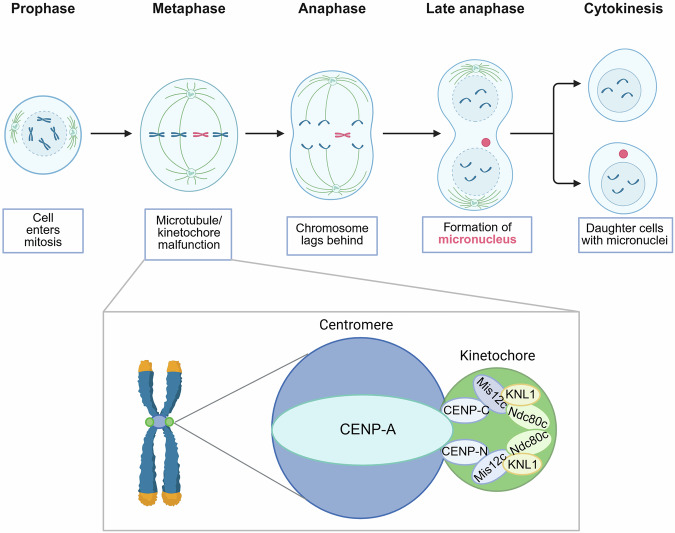
Mitotic defects leading to micronucleus formation. Chromosomes with incorrectly assembled kinetochores in the M phase are unable to attach to microtubules and lag behind other chromosomes, and they are also unable to partition correctly and equally into daughter cells after cell division, where they are encapsulated by the nuclear membrane at the end of the division and exist as micronuclei in the daughter cells (created in https://BioRender.com)

Mitotic defects are key initiators of micronuclei formation as they induce lagging chromosomes which provides the material basis for micronuclei. Some chromosomes fail to align at the equatorial plate during cell division and become delayed. This often indicates a defect in cellular kinetics, such as impaired assembly of the centromere-kinetochore complex.^28^ Under normal conditions, proper centromere-kinetochore assembly ensures the alignment of sister chromatids at the spindle’s equatorial plate, allowing a smooth transition into anaphase.^29^ As a highly precise and regulated process (Fig. 2), centromere-kinetochore assembly begins with the accurate deposition of centromeric protein A (CENP-A), which is a specialized histone variant that marks the centromeric DNA region and establishes the foundation for kinetochore assembly.^30^ Next, centromere-specific proteins, such as CENP-C and CENP-N, gradually accumulate at the centromere to form the inner kinetochore, stabilizing the chromosome-kinetochore connection. As the inner kinetochore develops, the outer kinetochore begins to assemble, which involves the recruitment additional protein complexes, including the Ndc80 complex, Mis12 complex, and Knl1.^31,32^ This process involves the recruitment of additional protein complexes, such as the Ndc80 complex, Mis12 complex, and Knl1, which provide structural support to the kinetochore and interact with spindle microtubules to ensure proper chromosome alignment and movement during cell division.^33^

Moreover, the kinetochore also plays a critical role in ensuring that chromosomes are correctly aligned and properly attached to microtubules.^34^ Spindle assembly checkpoint, through its interaction with the kinetochore, continuously monitors the status of kinetochore-microtubule attachment. Only when all chromosomes are correctly attached will the cell proceed to anaphase, thereby preventing chromosome misalignment and unequal segregation.^35^

Given the precision of this process, even minor errors can result in defective chromosome segregation, leading to the formation of lagging chromosomes. For instance, an improper concentration of CENP-A specific can disrupt the current attachment of sister chromatids to the equatorial plate, causing the missegregated chromosomes to remain in the cytoplasm and be encapsulated by the nuclear membrane to form micronuclei.^36,37^ Research has also identified the emergence of a large number of nanonuclei, a special form of micronuclei, during the formation of human artificial chromosomes (HACs) with CENP-A ( − ).^38^

Overall, lagging chromosomes arise as a consequence of abnormal mitosis in abnormal mitotic dynamics. This dysfunction involves not only defective centromere-kinetochore assembly but also epigenetic alterations that affect mitotic progression.

### DNA double strand breaks lead to micronucleus formation

Unlike mitotic defects, which usually result in an entire intact chromosome being wrapped into micronuclei, micronuclei formed by DSBs usually contain chromosome fragments.^39,40^ Typically, the DNA damage response (DDR) is activated when DSB occurs in the interphase, which initiates cell-cycle checkpoints via ATM and ATR, halting the cell cycle at the G1/S or G2/M transition until DNA repair is completed.^41^ In contrast, when DSBs occur during mitosis, they do not arrest cell cycle progression since cells prioritize completing division over repairing damage, which leads to micronuclei formation.^42–44^ In addition, the two primary DSBs repair pathways, homologous repair (HR) and non-homologous end-joining (NHEJ), exhibit phase-dependent activity: HR is predominantly active during interphase, whereas NHEJ appears to be inhibited in mitosis.^45–47^

The chromosome breaks into two segments: an acentric fragment and a centric fragment. The acentric fragment, lacking a centromere, usually wanders into the cytoplasm and is randomly assigned to one of the daughter cells, where it ultimately forms a micronucleus.^42^ Theoretically, the centric fragment could still participate in mitosis like any other normal chromosome and be randomly distributed to the daughter cells. However, due to its exposed DNA ends, it may undergo with another chromosome during the next cell cycle, resulting in the formation of a bicentric chromosome.^48,49^

The formation of dicentric chromosomes is a key feature of the break-fusion-bridge cycle.^50^ During a subsequent mitotic division, the dicentric chromosome is elongated as the daughter cytoplasm gradually separates, forming a chromatin bridge between the two daughter cells. This bridge is severed by some factors such as mechanical stress produced by actin or three-prime repair exonuclease 1 (TREX1). The residual bridge chromatin fragments are randomly distributed between the two daughter cells, and some fragments become encapsulated by the nuclear membrane during the next mitosis to form micronuclei.^51,52^

In addition to lagging chromosomes and DSBs, telomere damage also contributes to micronuclei formation. Still, it also repeats the BFB cycle after DSB and is therefore not a direct cause of micronucleus formation.^53^

### Nuclear buds induce micronucleus formation

Nuclear buds are nuclear protrusions that arise when the nucleus expels excess DNA fragments, including those generated through the break-fusion-bridge cycle or aneuploidy.^25^ Morphologically, nuclear buds closely resemble micronuclei; however, unlike micronuclei, which exist independently of the primary nucleus, nuclear buds remain connected to the nucleus through chromatin.^54^ Nuclear buds often appear in conditions that promote gene amplification and accompanied by folate deficiency. In vitro studies using mammalian cells have shown that amplified DNA is located in specific areas around the nucleus and cleared through nuclear budding during the interphase of the cell cycle.^55^ Moreover, evidence suggests that micronuclei have can originate from nuclear budding after ionizing radiation. The irradiated cell nucleus is filled with Rad51 protein complexes and aggregates into lesion clusters. After binding with damaged DNA, it is expelled from the nucleus as nuclear buds and subsequently forms micronuclei.^56,57^ Unlike micronuclei formed from lagging chromosomes, those arising from nuclear budding during the interphase lacks lamin B in their nuclear membrane—a characteristic also observed in micronuclei generated by chromatin bridge cleavage.

In addition, several extracellular non-genotoxic factors appear to induce nuclear bud formation. Fresh serum has been reported to induce nuclear budding as it is rich in serum response factors, which regulate actin contraction.^58,59^ Nuclear budding appears to be pulled out of the nucleus in a serum-rich environment. However, this effect is not observed in normal cells, as excessive serum levels lead to the formation of large cytoplasmic vesicles instead of nuclear bubbles.^27^

### The presence of micronuclei-inducing genes

It remains difficult to fully explain the precise mechanisms by which various factors contribute to micronuclei formation. To elucidate these mechanisms, it may be more effective to consider intrinsic susceptibility factors within the cells.

Recent research has provided groundbreaking insights into the hereditary factors that regulate micronuclei formation.^60^ In a comprehensive study analyzing up to 997 mutant models of mice, 145 genes (71 negative and 74 positive) were identified as being associated with micronuclei formation. Among these, cells with the deletion of DNA Replication and Sister Chromatid Cohesion 1 (DSCC1) gene, which plays a crucial role in DNA replication and sister chromatid adhesion during S-phase, resulted in the most significant increase in micronuclei.^60,61^ The association between DSCC1 loss and increased micronuclei formation is particularly noteworthy.

The presence of micronuclei-related genes in the body has been implicated in a range of human diseases, such as DSCC1-deficient abnormalities of bone development. However, the extent to which such diseases are associated with micronuclei formation remains largely unknown. These findings reveal the clinical potential of micronuclei, and it is foreseeable that targeting micronuclei to intervene in disease progression may become a reality.

## The fates of micronuclei

**Fig. 3:**
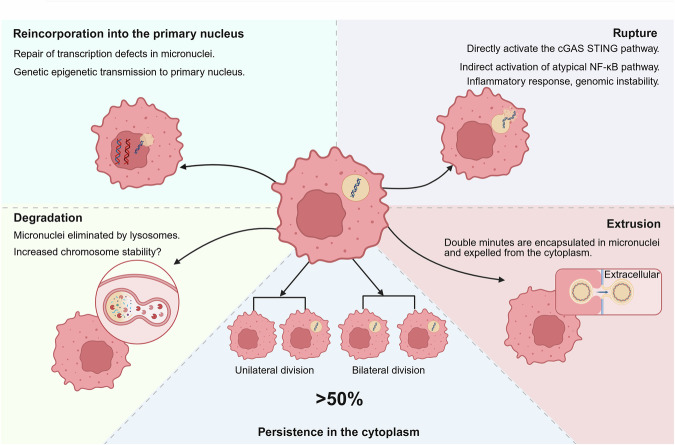
Different fates of micronuclei. Reincorporation into the primary nucleus repairs transcriptional defects and may facilitate the transfer of genetic information to the prokaryotic nucleus. Degradation involves lysosomal elimination of micronuclei, potentially enhancing chromosomal stability. Micronuclei rupture activates cGAS-STING and atypical NF-κB pathways, leading to inflammatory responses and genomic instability. Squeezing encapsulates two minutes within micronuclei and expels them from the cytoplasm. Over 50% of micronuclei persist in the cytoplasm and undergo unilateral or bilateral divisions during subsequent mitotic cycles (created in https://BioRender.com)

Micronuclei undergo several fates after formation: reincorporation, persistence, rupture, degradation, and extrusion (Fig. 3). These outcomes affect the host cells in various ways, making it essential to investigate the underlying mechanisms underlying.^62^

### Reincorporation into the primary nucleus

The first direct observation of micronuclei reincorporation was reported in the 1990s by Rizzoni et al., who aimed to prove the mitotic indirect non-disjunction (MIND) hypothesis. This hypothesis proposes that chromosomes within micronuclei can undergo simultaneous mitotic divisions with the primary nucleus during division. Their findings were surprising, as they not only demonstrated the existence of reincorporation but also identified it as a source of aneuploidy in Vicia faba and human lymphocytes.^63,64^ However, since reincorporation occurs during mitosis, it is difficult to determine (or distinguish?) whether the micronuclei remaining after division were initially present or formed from new chromosome segments.^62,65,66^ This limitation was later addressed with the use of live-cell imaging, which enabled direct observation of the intracellular trajectory of micronuclei chromosomes.^67^

Reincorporated micronuclei primarily contain chromosome segments derived from lagging chromosomes and centric fragments, since these elements are more likely to participate in subsequent mitosis.^65^ While reincorporated micronuclei chromosomes resemble intact chromosomes as they contain metacentromeres, they still often exhibit functional defects, such as reduced transcription levels.^7^ Notably, some daughter cells originating from micronuclei-containing cells have exhibited low transcription levels. Division tracking showed the reincorporation of micronuclei chromosomes, with transcriptional defects in daughter cells mirroring those in the micronuclei-containing mother cells, indicating that such functional defects in micronuclei chromosomes may be inheritable through the primary nucleus.^68^ Fortunately, this phenomenon does not occur universally. In most cases, micronuclei chromosomes recover their transcriptional function after reincorporation, with only those that have sustained extensive DNA damage passing defects to daughter cells.^68^

Micronuclei containing acentric fragments or kinetochore-deficient segments exhibit distinct fates. If these chromatin segments condense into chromosomes during prophase, they tend not to be reincorporated into the primary nucleus but exist as cytoplasmic chromosome segments after nuclear membrane rupture. Consequently, these fragments can form micronuclei or multiple micronuclei by the end of the cell cycle. However, even when reincorporation occurs, these segments may be missegregated into daughter cells during subsequent divisions. It is speculated that kinetochore-deficient segments may have a lower probability of missegregation than acentric segments, as some degree of kinetochore repair may occur after reincorporation.^69^ However, further experimental evidence is required to confirm this hypothesis.

### Persistence in the cytoplasm

Although micronuclei can follow various fates, most research has focused on membrane rupture. However, a large proportion of micronuclei persist in the cytoplasm or are inherited by daughter cells after division. Over half (62%) of micronuclei exist in cancer cells for over one cell cycle.^40^ In mouse embryos, the vast majority ( >90%) of cells containing micronuclei pass them on to a daughter cell, a phenomenon termed unilateral inheritance. Cayetana et al. proposed that the functional kinetochore is lost when the lagging chromosome is encapsulated within a micronucleus, preventing proper distribution to both daughter cells in the next mitosis despite the presence of a centromere.^70^ Whether unilateral inheritance of micronuclei occurs as a passive or active process remains unresolved, though current evidence suggests it is random.

One possible explanation for micronuclei’s persistence is that the centromeres of the chromosomes contained in the micronuclei are damaged. During anaphase, the micronuclei chromosomes that failed to recruit to the equatorial plate remain in the cytoplasm due to their inability to participate in mitosis. Similar to micronuclei formation, the assembly defect of the kinetochore is one of the main contributors to ineffective chromosomes. For example, some micronuclei chromosomes lack spindle assembly checkpoint due to the inability to recruit Aurora-B and Mad1 proteins, making normal mitosis impossible.^71^ Additionally, the defects in the nuclear pore complex of micronuclei prevent them from recruiting the required methylase, which is crucial for proper kinetochore assembly.^69^ In addition to these chromosomes containing damaged centromeres, some chromosome fragments isolated into micronuclei completely lack centromeres.^72^ The widespread persistence of micronuclei makes it reasonable to consider them a non-random process, though the precise underlying mechanisms require further investigation.

The persistence of lamin (−) micronuclei in the cytoplasm is a unique phenomenon. Theoretically, the envelope of lamin (−) micronuclei is frequently disrupted by replication stress signals from the primary nucleus or cytoplasmic actin during mitosis. Consequently, these micronuclei struggle to maintain their integrity throughout the replication process. Asynchronous DNA replication between micronuclei and the primary nucleus explains why lamin (−) micronuclei often fail to survive. Lamin protects the nucleus from cytoplasmic stress and other destabilizing factors. In the absence of lamin, micronuclei receive cell division signals may be unable to replicate due to nuclear pore complex defects and improper assembly of replication proteins, resulting in DNA damage. However, micronuclei do not rupture quickly, and releasing damaged DNA may occur gradually. Therefore, micronuclei that lacks lamin and have not yet undergone rupture can persist in the cytoplasm for multiple cell cycles, although they can be delivered to only one daughter cell.^73,74^ The proportion of micronuclei following this trajectory remains unclear and requires further investigation.

### Rupture

Micronucleus rupture has been observed in a variety of cell types, leading to the realization that membrane fragility and instability are intrinsic properties of micronuclei rather than of their cell species.^4^ Notably, rupture does not imply the instant destruction of micronuclei, it merely signifies the loss of membrane integrity. This section primarily reviews the structural and functional basis for the rupture of micronuclei and its consequences, particularly the exposure of their internal chromosomes.

#### Composition of micronucleus membrane

In eukaryotic cells, the genome is enclosed within a double-layered lipid membrane consisting of the inner nuclear membrane and outer nuclear membrane. These two membrane are interconnected by the nuclear pore complex.^75^ Lamin is located in the inner nuclear membrane and has three types: lamin A, lamin B (including B1 and B2), and lamin C. The primary function of lamin is to reduce mechanical stress on nuclear membrane and chromosomes, thereby preserving membrane integrity and chromosome stability.^76^ Structurally, the micronuclear membrane closely resembles the primary nuclear membrane in terms of its composition. However, there is a significant difference in the relative proportions of its components, which significantly contributes to the fragility of the micronuclei membrane. Notably, lamin levels in micronuclei membranes are markedly lower than those in the primary nucleus, which directly contributes to the low compressive strength of the micronuclei membrane.^20^

Generally, the sequence of nuclear membrane rupture follows a sequential process. It begins with the appearance of weak points in the membrane. When micronuclei are subjected to external damaging factors (such as mechanical stress), the weak points are the first to rupture, allowing chromatin to escape in a process similar to hernia formation.^76^

#### Micronucleus membrane rupture mechanism

The absence of lamin B is a significant factor contributing to micronuclei rupture. During late mitosis, core nuclear envelope proteins aggregate around chromosomes and are subsequently distributed near the spindle. Meanwhile, nuclear pore complex components and non-core proteins remain in the chromosome outer region away from the spindle until mitosis is complete, after which these proteins slowly assemble during interphase.^77–79^ Spindle microtubules inhibit the recruitment of nuclear envelope, nuclear pore complex, and non-core proteins to lagging chromosomes, resulting an exceptionally high concentration of the core nuclear envelope proteins within these chromosomes. Due to the absence of nuclear pore complex, micronuclei exhibit defects in the nuclear-cytoplasmic transport, impairing the import of essential proteins such as DNA replication and repair proteins and proteins required for nuclear envelope integrity (including lamin B).^80^ When external mechanical stresses (e.g., actin-mediated nuclear compression) target weak regions of the micronuclei envelope lacking lamin B, nuclear envelope rupture occurs promptly. Even in the absence of actin, the rupture of lamin-deficient nuclear envelope cannot be reversed. Although the role of actin in micronuclei membrane rupture remains controversial, one possible explanation is that most lamin-deficient micronuclei membrane, especially those in smaller micronuclei, cannot persist through interphase and would then be destroyed by actin. However, it has been confirmed that mechanical forces generated by actin can promote the formation of chromatin hernias.^81,82^ Therefore, we hypothesize that the leading role of mechanical stresses may focus on micronuclei formation rather than rupture.

The size of the micronuclei also appears to correlate with its susceptibility to rupture. Smaller, highly curved micronuclei exhibit higher cGAS levels and lower lamin B1 levels than larger, flatter micronuclei.^83^ Although the underlying mechanism is unclear, high membrane curvature seems to have significant deficiencies in recruiting lamin B1 and some other nuclear proteins, which may explain why smaller micronuclei are more prone to rupture.

Besides lamins as an assembly factor and mechanical stress as a destructive factor, chromosome length and gene density have also been identified as factors influencing micronuclei membrane rupture.^84^ The sizes of micronuclei correlates directly with the lengths and numbers of chromosomes. Even in the absence of lamin B1 and nuclear pore complex, the gap in micronuclei membrane can still be maintained at a low level as long as it possesses a high gene density. Unfortunately, despite its stabilizing effect, high gene density does not prevent the rupture of micronuclei membrane. The observation may explain the increased stability of micronuclei in DLD-1 cells, which lack segment gene sequences.^84,85^

**Fig. 4:**
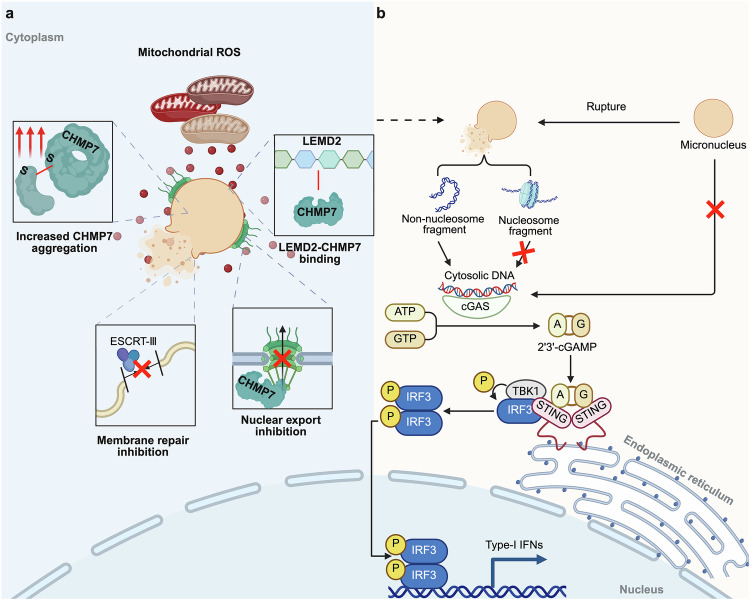
Precursors and consequences of cGAS activation by micronuclei. **a** Mechanism of mitochondrial ROS-promoting micronuclei membrane rupture. **b** Micronuclei rupture releases non-nucleosome fragments into the cytoplasm, where they bind to cGAS and produce cGAMP. cGAMP subsequently binds to STING proteins in the rough endoplasmic reticulum (RER), inducing a conformational change in STING and recruiting TBK1 and IRF3. This results in IRF3 phosphorylation, which forms a dimer that translocates to the nucleus and contributes to IFN-I secretion (created in https://BioRender.com)

Recently, studies by Bakhoum et al. and Santaguida et al. have revealed an endogenous mechanism by which mitochondrial ROS mediates micronuclei membrane rupture via CHMP7 (Fig. 4a). On the one hand, mitochondrial ROS inhibitXPO1-dependent CHMP7 nuclear export, leading to a massive accumulation of CHMP7 in micronuclei. This aberrant accumulation promotes CHMP7 binding to LEM domain nuclear envelope protein2 (LEMD2), triggering the collapse and rupture of micronuclei membrane. On the other hand, ROS-induced cysteine oxidation promoted CHMP7 clustering, reducing its interaction with other members of the endosomal sorting complex required for the Transport III (ESCRT-III) complex.^86,87^

#### Membrane repair after rupture

Cells possess a robust nuclear membrane repair mechanism during interphase facilitated by active nuclear envelope assembly. The repair mechanism, involving the recruitment of repair proteins from the endoplasmic reticulum, fills the gaps in the nuclear membrane within a remarkably short timeframe—ranging from a few minutes to a few hours.^88,89^ Similar to the nuclear envelope assembly during mitosis, the barrier to autointegration factor (BAF) protein in the cytoplasm rapidly aggregates at sites of membrane rupture, recruiting the N-terminal domain (NTD) protein of LEM, lamin A, and ESCRT-III to the rupture site. Additionally, BAF binds to chromatin exposed due to membrane rupture, protecting chromatin from detection and destruction by cGAS.^90,91^

However, such nuclear envelope repair mechanism typically does not occur in micronuclei. Although BAF and ESCRT-III are detected in micronuclei following membrane rupture, the small size of the micronuclei limits CHMP7 aggregation, unlike that in the primary nucleus.^52^ Not only will the accumulatedCHMP7 fail to interact with other ESCRT-III components to exert membrane repair, but also continue to signal the need for micronuclei membrane repair, leading to an infinite accumulation of ESCRT-III. This persistent recruitment results in extensive deformation and further micronuclei membrane rupture.^87,92^ Therefore, the role of ESCRT-III in micronuclei is dual-faceted; while essential for repair, it can also exacerbate micronuclei rupture if it is not accurately localized to the membrane.^87,92–94^ For example, ROS recruits the autophagy receptor P62 within the micronuclei, resulting in a reduction in CHMP7 levels by autophagy. Consequently, ESCRT-III cannot be directed to the injury site, impeding damage repair and promoting micronuclei rupture. Therefore, the critical membrane concentration of ESCRT-III is crucial for genomic stabilization of micronuclei.^87^ Therefore, the critical membrane concentration of ESCRT-III is important for the genomic stabilization of micronuclei. Additionally, the localization of TREX1 in the micronuclei appears to rely on the recruitment effect of BAF or the remodeling effect of ESCRT-III. However, TREX1 actively degrade micronuclei DNA, which limits the activation of cGAS to some extent.^52^ This is similar to the function of BAF, while raising a new question: does BAF directly inhibit cGAS, or is this the result of its recruitment of TREX1 to degrade DNA?

#### The consequences of micronucleus membrane rupture

One of the most well-recognized consequences of micronuclei membrane rupture is the activation of the cGAS-STING pathway. Hyperactivation of the cGAS-STING pathway is one of the causes of autoimmune disease, as the exposure of cytoplasmic DNA following micronuclei rupture activates the pathway and triggers an immune response. However, not all intact micronuclei are cGAS-negative, nor do all ruptured micronuclei necessarily activate cGAS. For instance, if an intact micronuclei contain a sufficient amount of Importin-β—a key nuclear pore complex component required for cGAS nuclear translocation—it may still exhibit cGAS-positive localization.^95^ However, the distribution of cGAS foci differs: those in intact micronuclei are strictly mild and diffuse, whereas cGAS in ruptured micronuclei accumulates in distinct, brightly separated foci.^96^

Notably, controversy remains over the activation of the cGAS-STING pathway by micronuclei. Patrick et al. first proposed that it is chromatin bridges, rather than micronuclei, that activate cGAS after mitotic errors, even though micronuclei recruit cGAS. Compared to micronuclei, chromatin bridges expose a greater amount of dsDNA, making them more potent activators of cGAS, though this is not absolute.^97^ In addition to DNA exposure levels, other factors also influence the recruitment of cGAS, such as histone modifications (e.g., H3K79me2) of the chromatin before encapsulation by the micronuclei membrane. Furthermore, micronuclei with high transcriptional activity also seem to have a strong repulsive effect on cGAS localization.^98^ Another rigorous experiment demonstrated that activation of the cGAS-STING pathway after γ-irradiation was unrelated to micronuclei, as STING remained activated regardless of the presence or absence of micronuclei in the cells, which appeared to result from mitochondrial DNA (mtDNA) release.^99^ A recent study further provided a plausible explanation for micronuclei rupture without cGAS activation: acidic patches on histones H2A and H2B inhibit cGAS activation, thereby preventing nucleosome-containing micronuclei from triggering the cGAS pathway.^100^ This suggests that incomplete nucleosome assembly may allow other forms of self-DNA releases, such as mtDNA leakage, to serve as alternative cGAS-STING pathway activators.^100,101^ These emerging studies challenge the long-standing assumption that micronuclei universally activate cGAS while simultaneously refining the understanding of self-DNA sensing mechanisms. We suggest that micronuclei remain an effective activator of cGAS, but only when the micronuclei membrane is ruptured and contain chromosomes that lack nucleosomes (Fig. 4b).

#### Cytoplasmic chromosome fragments (CCFs) and cGAS activation

Two recent studies have revealed potential mechanisms involved in the activation of cGAS by cytoplasmic CCFs. First, the MRE11-RAD50-NBN complex binds to nucleosomes, releasing cGAS from acid patch-mediated isolation and thereby enabling it to bind dsDNA.^102^ Second, the DNA-dependent protein kinase catalytic subunit (DNA-PKcs) enhances nucleosome sequestration of cGAS, thereby suppressing the activation of the cGAS-STING pathway.^103^ A new question thus arises as to whether such micronuclei can still be classified as “nuclei” and that, in essence, it will not be micronuclei that activate cGAS but rather CCFs. Similarly, cGAS can localize to chromosomes that have not yet been encapsulated into micronuclei following improper segregation during mitotic exit, raising further ambiguity regarding whether cGAS activation is riven by micronuclei or by CCFs.^104^

The activation of cGAS-STING triggers distinct reactions depending on the triggering factors. Specifically, when triggered by the rupture of the primary nucleus membrane, this pathway promotes the secretion of senescence-associated secretory phenotypes (SASP).^105^ When triggered by exogenous invading DNA, such as that from bacteria, viruses, or mtDNA, it induces the expression of immune and inflammatory mediators, including type I interferon (IFN-I).^106^ In the context of micronuclei membrane rupture, activation of cGAS occurs predominantly in tumor cells. Persistent micronuclei rupture in cancer cells inhibits IFN and activates atypical NF- κB, shifting the function of cGAS-STING from tumor immunity to the promotion of STING-dependent invasion and metastasis of cancer cells.^107–109^

As research continues to expand, the understanding of the complex relationship between micronuclei and the cGAS-STING pathway is increasingly evolving. Ongoing studies will provide a clearer picture of the molecular mechanisms underlying self-DNA sensing and immune activation in the future.

### Degradation

Autophagy is an intracellular “self-digestion” process that degrades damaged organelles and proteins.^110^ In cells with increased micronuclei frequency, the autophagy marker LC3 is elevated, and a portion of micronuclei co-localizes with LC3, indicating micronuclei membrane degradation and the presence of DNA damage (γH2AX + ) markers.^111,112^ Two types of autophagy are classified based on the size of the autophagic target—macroautophagy and microautophagy. While macroautophagy contributes to organelle quality control, its role in targeting nuclear degradation? remains to be fully elucidated.^111,113^ In yeast, the primary distinction between macroautophagy and microautophagy lies in the nuclear vesicle junction (NVJ)—a unique structure between the vacuole membrane and the nuclear membrane, which is exclusive to microautophagy. Current evidence suggests that small micronuclei (diameter 350–390 nm), located outside the NVJ, are degraded via macroautophagy, while micronuclei-like structures (diameter 580–770 nm) within the NVJ undergo microautophagy.^114^

Beyond activating inflammatory responses, cGAS has also been implicated in independently mediating autophagy in micronuclei. Structurally, cGAS contains five LC3-interacting regions (LIRs), which bind directly to LC3. cGAS functions as a receptor to mediate autophagy in micronuclei, promoting their clearance via an ATG14- and ATG7-dependent classical autophagy pathway.^115^ This mechanism operates independently of STING, thereby bypassing IFN-I responses, as confirmed in the liver cells of cGAS-deficient mice.^116^

However, the autophagic clearance of micronuclei has not been linked to any significant consequences, as it primarily functions as a degradative pathway without established downstream consequences (check if this is correct). Further investigation is needed to determine the broader implications of autophagy-mediated micronuclei clearance. Notably, the relatively low levels of autophagy associated with micronuclei degradation present a substantial challenge for further research in this area.

### Extrusion

Micronuclei extrusion refers to the process by which micronuclei are expelled from the cell to the outside of the cell, with distinct biological significances in different cell types. It was first proposed in erythrocytes, where micronuclei are expelled during maturation, similar to the primary nucleus, with most extruded micronuclei containing intact chromosomes.^117^ However, whether micronuclei are actively eliminated through extrusion remains uncertain, as their extrusion has not been clearly observed in live cell imaging.^74^

In primate embryonic cells, micronuclei extrusion functions as a self-protection mechanism. During embryonic cell lysis and proliferation, chromosomal missegregation often generates CCFs, which are rarely reintroduced into the primary nucleus. Instead, embryonic cells encapsulate CCFs into micronuclei and expel them from the cell.^118^

In tumor cells, double minutes (DMs)—a type of cyclic ecDNA typically found in cancer cells—are selectively incorporated into micronuclei and subsequently expelled. This process mitigates the effects of partial oncogene amplification within DMs, thereby restoring the tumor cell phenotype. Current research has attempted to target the ErbB1 gene in A549 cells via micronuclei extrusion to improve therapeutic outcomes in non-small cell lung cancer (NSCLC).^119,120^ Interestingly, DMs-rich micronuclei expelled by cancer cells retain a normal envelope, and their internal DNA does not undergo extensive degradation.^119^ Although this contrasts with micronuclei extrusion in embryonic cells, it suggests that both intact and incomplete micronuclei can be expelled or that micronuclei extrusion occurs as a random and unregulated process.^118^

## Chromothripsis

Chromothripsis is a unique form of genomic instability characterized by dozens to hundreds of DNA DSBs occurring on a few chromosomes, followed by random reassembly during repair.^21^ Although chromothripsis is not strictly a fate of micronuclei, it is one of the most extreme consequences of micronuclei formation. A recent review provides a detailed overview of recent advancements in chromothripsis research.^121^

**Fig. 5:**
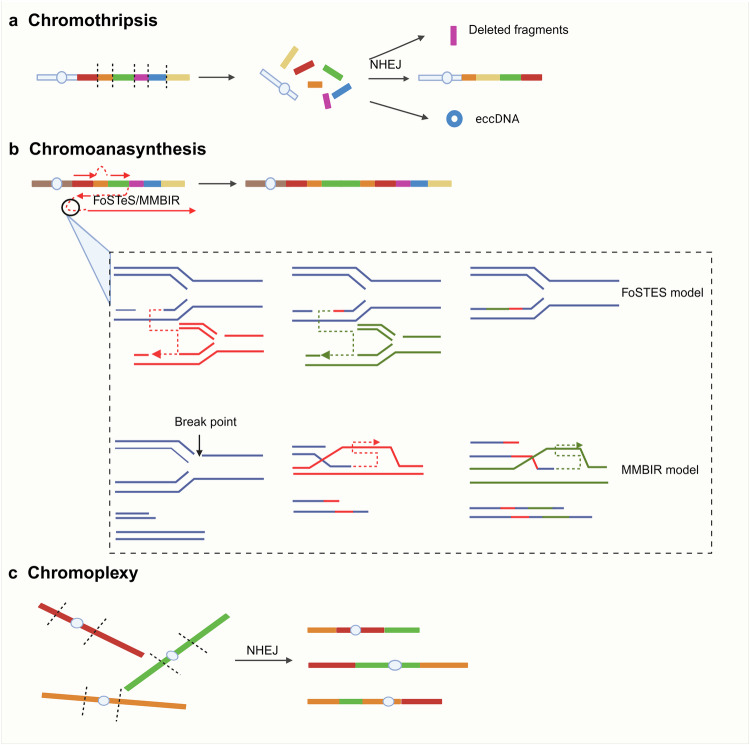
Three extreme chromosomal rearrangements categorized as chromoanagenesis. **a** Chromothripsis: Localized chromosomal shattering generates hundreds of clustered DNA breaks. Fragments randomly reassemble via error-prone NHEJ, causing massive rearrangements, oscillating copy numbers, and frequent loss of interstitial segments. Detached DNA may circularize into eccDNA; **b** Chromoanasynthesis: A replication-repair process where stalled/broken forks disengage from the original template and fuse with proximal forks. Two subtypes: the fork stalling and template switching (FoSTeS): the lagging strand iteratively switches templates via microhomology, hopping between stalled/active forks before rejoining the original chromosome; the microhomology-mediated break-induced replication (MMBIR): broken forks use 2-15 bp microhomology to cycle through iterative dissociation-reassociation with new templates, resolving replication stress; **c** Chromoplexy: Cross-chromosomal breakage-repair networks create chimeric chromosomes. Transcription-induced topological stress initiates break; some undergo NHEJ-based random ligation, others remain unrepaired, forming chain-like structural patterns; **c** Replication Restart Mechanisms (Created in https://BioRender.com)

For a long time, it was believed that genetic alterations within tumor cells accumulated gradually over many years or even decades. However, the natural rate of nucleotide mutations and chromosome rearrangement in cells is insufficient to generate the high number of mutations required to form tumor cells.^122^ Therefore, a mechanism capable of inducing large-scale genomic rearrangements within a short time frame is necessary to explain the high mutation rate in tumor cell genes. Over a decade ago, a novel chromosomal rearrangement pattern was identified in a patient with chronic lymphocytic leukemia (CLL), which was later known as “chromothripsis”.^21^ Since its discovery, chromothripsis has been recognized as a distinct form of complex chromosomal rearrangement and is now more accurately classified under the broader category of “chromoanagenesis.”^123^ Chromoanagenesis describes the state of chromosome shattering within a single cell cycle and encompasses three major processes: chromothripsis, chromoplexy, and chromoanasynthesis, with chromothripsis representing the most extreme case (Fig. 5).

While the precise origin of chromothripsis remains unclear, the micronuclei hypothesis is considered to be one of the most reliable mechanisms. As a marker of CIN, micronuclei no longer act merely as an indicator of DNA damage; rather, it has served as a driving force of the formation of DNA lesions.^124–126^ In addition, other proposed mechanisms, including telomere crisis and break-fusion-bridge, have also been used to explain the massive, clustered genomic rearrangements.^53,127,128^ Notably, these mechanisms are centered around micronuclei, as both telomere crisis and the break-fusion-bridge cycle involve processes that lead to chromosome fragmentation.

### Micronuclei directly induce chromothripsis

In summary, DNA damage in micronuclei is closely related to chromothripsis. On one hand, most micronuclei DNA damage occurs during interphase—the micronuclei membrane rupture and releases DNA into the cytoplasm, where it undergoes chromosome fragmentation and random recombination and ultimately leading to chromothripsis. On the other hand, abnormal replication of micronuclei chromosomes during mitosis can also result in damage through various mechanisms.^20,51,129^

Since micronuclei contain only one or a few chromosomes, their physical separation from the primary nucleus explains why large-scale rearrangements induced by chromothripsis is confined to a limited number of chromosomes.^130^ Beyond structural damage caused by the micronuclei membrane rupture, the genetic material within micronuclei often deviates from a normal DNA structure. A significant amount of genetic material in micronuclei exists as DNA-RNA heterozygotes (R-loops) rather than double-stranded DNA.^129^ Adenine deaminase acting on RNA (ADAR) can bind to DNA-RNA hybrids within the micronuclei and edit them, generating deoxyinosine, which is subsequently converted into an ablation site by DNA base excision repair (BER) glycosylase and N-methylpurine glycosylase (N-MPG). This site can then be cleaved by BER endonuclease and apurinic/apyrimidinic (AP) endonuclease, creating a single-stranded DNA (ssDNA) gap. During DNA replication, these gaps can form DSBs, and DNA fragmentation via the DSB is a key step in chromothripsis. However, the mechanisms underlying DNA-RNA hybrid formation in micronuclei remains unclear and may be associated with abnormal transcription within these structures.^125^

We hypothesize that even if micronuclei are given sufficient time for DNA damage repair, the occurrence of chromothripsis remains inevitable. Therefore, micronuclei themselves do not possess a normal DNA repair mechanism, relying predominantly on the error-prone NHEJ pathway rather than the HP pathway that has a high repair accuracy. This contributes to the complex chromosomal rearrangements observed in chromothripsis.^131^

In addition, chromosomes in the micronuclei often exhibit insufficient or delayed replication compared to chromosomes in the primary nucleus. As early as 1970, an experiment fusing interphase and mitotic cells confirmed its ability to induce premature chromosome condensation (PCC) in interphase cells, and subsequent changes in interphase cells’ chromosomes are highly similar to those during chromothripsis.^132^ Now from a modern perspective, this research appears to simulate the process by which micronuclei drive chromothripsis outside the cellular environment. DNA replication in normal nuclei is often completed before the G_2_ phase, followed by chromosome condensation and preparation for entering the M phase. However, DNA replication within micronuclei proceeds at a slower rate, extending beyond the G2 phase and remaining incomplete when the cell enters mitosis. The division signal from the primary nucleus can trigger premature condensation and breakage of lagging chromosomes within the micronuclei. Subsequent studies have also supported the idea of PCC as a mechanism underlying chromosome breakage. Chromosome condensation generates mechanical stress at DNA damage sites. Incompletely replicated DNA within the micronuclei contains numerous damaged regions, hindering the DNA repair process and causing chromosome tearing. To resist this stress, human cells have evolved cell cycle checkpoints that allow sufficient time for DNA repair or replication to be completed. Unfortunately, DNA damage within micronuclei is often insufficient to activate these checkpoints, leading the cell cycle to a standstill.^40,133^

There is also an unexpected relationship between the spatial location of micronuclei within the cell and the extent of DNA damage. The mitotic driving protein kinesin family (Kif) member plays a crucial role in ensuring accurate chromosome separation during cell division. However, after knocking out Kif18a, cells still produce micronuclei with an intact nuclear envelope, but their chromosomes are positioned closer to the primary nucleus. Interestingly, these micronuclei do not exacerbate CIN or promote tumorigenesis.^134,135^ Conversely, for pre-existing micronuclei, those positioned further from the spindle of the cell center exhibits less damage in genetic material, while for chromosomes undergoing normal mitosis experience a higher probability of missegregation and micronuclei formation when located further from the spindles.^80,136^ These findings suggest that the more complete the membrane of the micronuclei, the more similar they are to the primary nucleus in terms of actual structural and functional aspects.

### Telomere crisis induces micronuclei formation and chromothripsis

Telomeres are protective fragments at the end of chromosomes in eukaryotic cells. Due to the inability of DNA polymerase to fully replicate the terminal regions of chromosomes, telomeres prevent the loss of important genetic information. With each cell division, the TTAGGG repeat sequences of telomeres progressively shorten until they reach a certain length, which triggers an aging state and stops dividing.^137^ The integrity of telomeres is a key factor in determining chromosome structure, and chromosomes with shortened telomeres are more involved in erroneous segregation compared to those with normal telomeres.

In healthy cells, telomeres are protected by a protein complex called shelterin, which binds to the TTAGGG repeat sequence. Shelterin components include telomere-binding proteins such as TPP1, TRF1, TRF2, TIN2, Rap1, and POT1. Prolonged cell proliferation leads to telomere shortening and the loss of shelterin protection. Typically, unprotected cells with critically shortened telomeres will age and die as a result. However, for cells carrying mutations of tumor suppressor genes such as P53 and RB, they continue to proliferate even with telomere grinding erosion. When the telomeres are fully eroded, sister chromatids may form end-to-end fusion, resulting in the formation of dicentric chromosomes^138,139^

Dicentric chromosomes persist throughout the entire mitotic process, and even after the physical separation of the daughter cells, they remain connected by a structure called chromatin bridges. These bridges do not break easily but gradually cleave over a period of 3-20 hours after the end of the previous round of mitosis. Before chromatin bridge dissociation, nuclear membrane rupture dissociation occurs, allowing the fragments to re-enter the nucleus and participate in the next cell cyle.^127,140^ This process is referred to as the telomere crisis, and the series of events following the telomere crisis—chromatin bridge formation—is known as the break-fusion-bridge cycle. However, not all chromatin bridges undergo gradual cleavage. Factors such as nuclease activity and transient actin mechanical forces can disrupt chromatin bridges, producing bridge-derived micronuclei that persist in the cytoplasm.^51^ Interestingly, micronuclei generated through telomere crisis do not always lead to the extreme result of chromosome division. Instead, they often activate the cGAS-STING pathway to induce cell aging and specific autophagy. In addition, cGAS can occupy telomeric sites, acting as a substitute for shelterin and thereby inhibiting end-to-end fusion—a key step in the break-fusion-bridge cycle.^141–143^

Therefore, a critical mediator is needed to disrupt bridge DNA, which in this case is TREX1. This enzyme typically degrades cytoplasmic-free DNA to resist inflammation.^144^ Similarly, when the micronuclei are exposed to their internal DNA due to the nucleus membrane rupture, TREX1 binds to the DNA and causes damage.^145^ TREX1 has also been proven to facility chromatin bridge dissociation, generating ssDNA by cleaving the dsDNA within the bridge, thereby inducing chromothripsis.^127^ This suggests that DNA damage has already occurred before the bridge breaks. Furthermore, TREX1 damages the exposed dsDNA in fragmented micronuclei. Notably, the telomere crisis alone is insufficient to trigger chromothripsis. Without TREX1, the genetic material alterations induced by telomere crisis are mainly simple rearrangements and break-fusion-bridge cycles rather than chromothripsis.^140^

In addition, telomeres are highly susceptible to oxidative damage, as their DNA sequence, rich in G sequences, is highly sensitive to reactive oxygen species (ROS). Notably, the most common DNA damage marker is 8-oxo guanine (8-oxoG). While telomeres are not sensitive to ROS-mediated 8-oxoG itself, cells lacking OGG1—the enzyme responsibly for8-oxoG clearance—experience long-term accumulation which increases replication pressure and accelerates telomere loss. This process significantly increases chromatin bridge and micronuclei formation.^6,146^ Additionally, the telomere damage caused by ROS seems unable to effectively activate the DDR, preventing appropriate repair of the damaged telomeres. Interestingly, although telomeric repeat binding factor (TRF1 and TRF2) exhibits low affinity for oxidatively damaged telomeres and telomerase has the capacity to degrade damaged telomeres, the damage caused by ROS is far more significant than this compensatory mechanism.^147,148^

### Chromothripsis drives oncogene amplification

As a catastrophic event, chromothripsis often leads to wide-spread cell death. However, cell death is not an inherent consequence of chromothripsis itself, but rather depends on which genes are affected. Similarly, the survival of cells following chromothripsis is also dictated by specific genetic alterations, which are not necessarily beneficial to the cell. It often amplifies oncogenes and mutations in tumor suppressor genes, which can be a more significant challenge for the body.

Stephens et al.^21^ first observed chromosome fragments in 1/4 of bone cancer cases when discovering chromothripsis. They also analyzed the copy number variations across 746 cancer cell lines, which revealed that approximately 2–3% of these cells exhibited rapid copy number changes, confined to a few chromosomes. These changes could not be explained by parallel rearrangements of different subclones, leading to the conclusion that chromothripsis occurs in at least 2–3% of cancer cells. This raises an important question: why is chromothripsis so frequently observed in cancer cells? Rausch et al.^149^ found numerous complex chromosomal rearrangements in Sonic Hedgehog medulloblastoma (SHH-MB) cells from a patient with Li-Fraumeni syndrome, a condition caused by mutations in the P53 tumor suppressor gene. Their findings determined that P53 mutations likely precede large-scale fragmentation and rearrangement. Based on these observations, they suggested that P53 mutations may either increase the susceptibility of cells to chromothripsis or provide resistance to cell death following chromothripsis. Additionally, when comparing CLL patients with and without chromothripsis, it was found that chromothripsis was associated with poor prognosis only in patients carrying P53 mutations. This indicates that, at least in CLL, abnormal P53—rather than chromothripsis itself— is the key to poor prognosis.^150^ Thus, the effect of chromothripsis on cells may be of secondary importance compared to events such as the P53 mutation, at least in terms of the results, which showed that P53-mutated CLL cells resisted the cytolethal effect of chromothripsis.

However, although many types of cancer have been found to carry fragments suspected of chromothripsis, the mechanism by which it drives tumor progression remains unknown. Extrachromosomal circular DNA (eccDNA), which originates from cyclized fragments of incorrectly separated chromosomes, has been proven to be a major driver of oncogene amplification in tumor cells. EccDNA not only facilitates the amplification of individual oncogenes but also generate powerful amplification sequences carrying multiple oncogenes.^22,151–154^ In pediatric neuroblastoma, chromothripsis-induced oncogene amplification has been documented, with a distinct mechanism known as “sequential amplification”: chromothripsis initiates eccDNA formation, followed by countless repeated pairing recombinations. The resulting amplification sequence either persists in the cytoplasm or integrates into the cancer cell genome, promoting tumor growth.^153^

Viewing chromothripsis solely as an indicator of poor prognosis in tumors is inaccurate. In fact, the absence of chromothripsis in some tumors does not necessarily result in a better prognosis than in those with chromothripsis. In neuroblastoma, MYCN gene amplification and ALK gene overexpression are commonly considered as well-established markers of poor prognosis in NB. However, the chromothripsis exerts an even greater impact on high-stage neuroblastoma, influencing the stability of neuronal growth cones and dysregulating Rac/Rho pathway regulatory factors. Interestingly, these subtypes with poor prognosis often do not carry amplification of MYCN.^155^ In patients with multiple myeloma, chromothripsis seems to be directly associated with poor prognosis and is one of the most common complex events, occurring in over 20% of patients. Chromothripsis is often an early event in multiple myeloma development, which can be unaffected by most other prognostic variables.^156–158^ However, chromothripsis does not always worsen the outcomes of osteosarcoma. Chromothripsis could lead to changes in genes such as p16lnk4a, which had previously been shown to drive osteosarcoma development after loss.^159^

In other cancers, chromothripsis exhibits bidirectional effects. In colon cancer and metastatic cancers, nearly all cancer cells in the samples displayed uneven chromothripsis events, leading to mutations in over 20 oncogenes, including APC, KRAS, and PIK3CA. Furthermore, each primary tumor and its metastatic counterpart displayed distinct chromothripsis patterns, reinforcing its role in colon cancer progression and metastasis.^160^ However, the cancer-promoting effect of chromothripsis is not absolute. A patient with complex karyotype familial adenomatous polyposis (FAP) showed that despite the occurrence of chromothripsis in the patient’s genome, a large-scale rearrangement caused the APC promoter to separate from its open reading frame (ORF), downregulating the expression of APC.^161^ Similarly, in gastric cardia adenocarcinoma, the prognostic impact of chromothripsis is related to the specific genetic characteristics. Focal amplification of the tyrosine kinase receptor 2 (ERBB2) gene is beneficial for patient survival, making it a positive prognostic marker in patients who have survived for more than two years. However, epidermal growth factor receptor (EGFR) amplification due to chromothripsis correlated with poor prognosis, further demonstrating the context-dependent effects of chromothripsis.^162^

An exceptional case of chromothripsis-driven benefit has been reported in a patient with WHIM syndrome, a genetic immunodeficiency caused by overexpression of CXCR4. As a receptor that controls the production and distribution of leukocytes, The aberrant CXCR4 copy is removed from hematopoietic stem cells by chromothripsis, revealing the potential of chromothripsis to cure the patient’s immune disorder.^163^ Although this is an exciting discovery, no other instances of disease remission due to chromothripsis have been documented. We infer that chromothripsis can be conceptualized as a high-risk genomic “lottery” with a significant elimination rate. Cells lacking essential survival factors (such as P53 mutations) are often eliminated following chromothripsis, whereas surviving cells are more likely to harbor pathogenic genetic alterations.

In general, the impact of chromothripsis remains unpredictable. It is unlikely to precisely determine which genes undergo what kind of recombination or what the consequences of these recombinations will be.

## Epigenetics and micronuclei

Abnormalities in the cell’s epigenetics may increase the formation of micronuclei, and the epigenetic landscape within micronuclei is receiving increasing attention.

### Epigenetic abnormalities drive micronucleus formation

DNA methylation is the only known epigenetic mechanism targeting DNA, primarily occurring at cytosine-phosphate-guanine (CpG) dinucleotides. In mitosis, hypermethylation of chromosome centromeres is crucial for normal chromosome segregation. For example, histone 3 lysine-4 dimethylation (H3K4me2) in centromeric chromatin is necessary for forming the HJURP-CENP-A complex, which is critical for kinetochore assembly. Hypomethylation of centromeric regions is linked to genomic instability. For instance, disrupting histone 3 lysine-9-trimethylation (H3K9me3) in heterochromatin around centromeres leads to defects in chromosome binding and segregation, causing gene rearrangements and micronuclei formation.^164–166^ In the epidermis, hypomethylation in keratinocytes deficient in the methyltransferase DNMT1 hyperactivates the cGAS-STING pathway. The underlying cause is defective kinetochore formation due to CENP hypomethylation, leading to numerous micronuclei activating cGAS.^24,167^ In addition to centromeres, high methylation on microtubule proteins is essential for normal cell division. Methylation is a post-translational modification (PTM) of dynamic microtubules. SETD2 is a histone methyltransferase responsible for the trimethylation of histone H3 lysine 36 (H3K36me3).^168^ Interestingly, subjects exposed to long-term chronic high-pollution environments instead showed lower frequencies of micronuclei. This is correlated with lower methylation of X-ray repair cross-complement 5 (XRCC5), which promotes chromosomal NHEJ repair.^169^

In addition to low methylation of DNA and histones, the deficiency of chromatin remodeling proteins plays a crucial role in micronuclei formation. SWI/SNF2 is a catalytic ATPase subunit of the chromatin remodeling complex, using ATP hydrolysis energy to move nucleosomes and reshape chromatin. The protein AT-rich interactive-domain 1 A (ARID1A) is the largest subunit of SWI/SNF, which participates in the formation of chromatin loops, promotes the accumulation of gamma H2AX in topologically associating domains (TADs), and transmits DSB signals, initiating the NHEJ and HR repair pathways. ARID1A deficiency can lead to an increase in micronuclei due to DSB repair defects.^170^ In cancer cells, one of the subunits of PBAF (a member of the SWI/SNF2 family), Polubromo1 (PBRM1), is often inactivated, leading to replication stress and increased micronuclei frequency in cancer cells. However, the inactivation of PBRM1 provides new opportunities for tumor treatment. The use of PARP inhibitors in such tumors can promote the cGAS-STING pathway, resulting in unexpected synthetic lethal effects.^171,172^ And lymphatic specific helicases (LSH) belonging to the SWI/SNF2 family also play a role in maintaining DNA hypermethylation; the absence of LSH often leads to comprehensive defects in genome methylation and a significant increase in micronuclei formation.^173,174^

Hypomethylation with increased micronuclei has been seen in clinical patients. Immunodeficiency-centromere instability-facial dysmorphism syndrome (ICF) is characterized by hypomethylation of satellite DNA (a special DNA sequence consisting of short, repetitive nucleotide sequences, some of which are distributed in centromeres and telomeres, and whose cytosine residues are usually highly methylated) in almost all tissues, and the centromeric heterochromatin of chromosomes 1, 9, 16, leading to sustained chromosome self-association and inducing micronuclei.^175^

### Epigenetic landscape in micronuclei

An elegant experiment induced and extracted micronuclei in two normal cells (human non-transformed breast epithelial cell MCF10A, RPE-1) and three highly malignant tumor cells (high-level serous ovarian cancer cell HGSOC, human triple-negative breast cancer cell MDA-MB-231, mouse triple-negative breast cancer cell 4T1), and found extensive defects in histone acetylation and ubiquitination. More importantly, most of the defects in histone PTMs in the micronuclei are caused by the rupture of the micronuclei membrane, while defects unrelated to the rupture appear before the formation of the micronuclei.^7^ A more emerging study applied five different genotoxic stress stimuli to the same type of cells: ionizing radiation (IR), Taxol, methylmethylsulfonate (MMS), hydroxyurea (HU), 5,6-dichloro-1-β-D-ribofuranosylbenzimidazole (DRB) to induce micronuclei. Although these stress stimuli can successfully induce micronuclei, the protein landscape within the micronuclei produced under different stimuli is not entirely the same. For example, the IR-induced micronuclei proteins reflect the most well-known micronuclei features: cGAS enrichment, transcriptional activity downregulation, and loss of chromatin remodeling proteins. It appears from the present that the epigenetic landscape and even the proteomic landscape within the micronuclei are determined by the genetic stimuli to which the cell is exposed, not by the cell type.^4^

The epigenetic defects in the micronuclei can be brought to the primary nucleus by reincorporation, leading to stable epigenetic abnormalities. Mechanistically, the complex composed of TOPBP1 and CIP2A binds to the micronuclei fragment and binds multiple fragments together, allowing them to enter the same daughter cell during division rather than randomly assigned to two daughter cells. This bolus rescued the loss of the micronuclei chromosomes, making reincorporation of the chromosome segments as a whole possible and reducing the occurrence of chromothripsis.^67,176,177^ Notably, although some micronuclei chromosomal damage sites can be repaired after micronuclei are incorporated into the primary nucleus, this does not imply a complete remission of CIN in micronuclei cells. Potential genomic changes in micronuclei will be brought to the primary nucleus upon reincorporation. For example, extensive loss of H3K9ac, H3K14ac, H3K27ac, etc. micronuclei lead to stable epigenetic abnormalities through reincorporation into the primary nucleus.^7^

## Micronucleus assays

The micronucleus assays were originally developed in the 1950s to assess the extent of DNA damage in plant cells by radiation.^13^ The principle is that micronuclei are colored by a dye that binds to DNA, so DAPI and Hoechst, commonly used for dyeing cell nuclei, can be used as stains. Except for the erythrocyte micronuclei assay, which is an RNA-based assay, Since the emergence of in vitro micronuclei assays, micronuclei assays have become one of the most popular methods for evaluating the genetic toxicity of various chemical or physical factors. However, although all micronuclei assays are based on the determination of micronuclei frequency, the cells, methods, and objectives used are not identical. Here, we have listed information on several of the most common micronuclei assays in the table, followed by a more detailed introduction (Table 3).

**Table 3:** Different types of micronucleus assays

Type of assays	Birth time	Cell used	Purpose	Characteristic
Cytokinesis-block micronucleus assay	1985	Peripheral blood lymphocytes of animals	Biological monitoring Genotoxicity assessment Biology experiments	The effect of cytokinesis on accuracy is excluded, and microscopic counting is convenient, but the addition of cytochalasin B is required, and the cost is high.
Erythrocyte micronucleus assay	1970s	Reticulocytes in animal bone marrow or peripheral blood	Biological monitoring Genotoxicity assessment	Convenient but less accurate, most suitable for patients after splenectomy
Buccal micronucleus assay	1980s	Human epithelial buccal cells	Biological monitoring Genotoxicity assessment Biology experiments Genotoxicity of lifestyle factors Large-scale human genetic survey	Simple and convenient for large-scale census but currently it is not a standard micronucleus assay
High-throughput micronucleus assay	2010s (predecessor appeared in the 1990s)	Animal red blood cells	Genotoxicity assessment Biology experiments	A large number of samples can be processed in a short period and can be scored directly based on the number of micronuclei, but requires instrumental support

### Cytokinesis-block micronucleus assay

Cytokinesis-block micronucleus assay is the most widely used method for micronuclei determination, as described by Schmid et al. in their research.^14^ Since micronuclei can only be observed after cell mitosis, it is not accurate to directly count the micronuclei of cells after injury stimulation without processing. Cytochalasin B is a cell-penetrating fungal toxin that can bind to the prickly end of actin, thereby inhibiting the elongation and shortening of actin filaments, preventing actin filaments from aggregating and forming microfilaments, ultimately leading to impaired cytoplasmic division. Cytochalasin B is a cell-penetrating fungal toxin that can bind to the prickly end of actin, thereby inhibiting the elongation and shortening of actin filaments, preventing actin filaments from aggregating and forming microfilaments, ultimately leading to impaired cytoplasmic division. Fortunately, cytochalasin B does not affect the division of the nucleus, so it does not hinder the production of micronuclei. When exposed to cytochalasin B, cells undergo mitosis and do not proliferate but instead form binucleated cells.^178^ Therefore, the number of cells does not increase after treatment with cytochalasin B, significantly improving the accuracy of micronuclei determination.

In the cytokinesis-block micronucleus assay, the most commonly used cells are peripheral blood lymphocytes, including humans and other animals (fish, primates, rodents, etc). Especially when the source of lymphocytes is humans, because the human lymphocyte cycle is not the same, the position of cytochalasin B is even more irreplaceable.^19,179,180^ More importantly, compared to other micronuclei assays, the cytokinesis-block micronucleus assay has a broader application in monitoring the genotoxic effects caused by occupational exposure factors. Although the reason is unclear, it may be because the cells used in the cytokinesis-block micronucleus assay are peripheral blood lymphocytes, which are less affected by lifestyle factors such as smoking and drinking. In addition, compared to red blood cells, lymphocytes are not easily destroyed and cleared by the spleen, so they can reflect longer-term and far-reaching stimuli because occupational exposure is often a long process. Currently, the cytokinesis-block micronucleus assay has been expanded to monitor the existing micronuclei in cells and measure markers such as nucleoplasmic bridges and nuclear buds, which is beneficial for a more comprehensive assessment of DNA damage.^181^

### Erythrocyte micronucleus assay

Compared to cytokinesis-block micronucleus assay, erythrocyte micronucleus assay was developed earlier and was the first method to employ animal cells for micronuclei detection. However, it was not widely adopted after its emergence because early erythrocyte micronucleus assays relied on reticulocytes extracted from rodent bone marrow, a process that was too expensive in terms of experimental time and economic cost.^14^ In addition, since flow cytometry was not widely used at the time, bone marrow samples often contained mast cells, neutrophils, and other interfering cell types, which caused significant interference in micronuclei determination.^182^

The introduction of acridine orange successfully overcame many of these limitations, enabling the use of peripheral blood erythrocytes for micronuclei testing. Normally, erythrocytes mature in the bone marrow before entering circulation. However, a small percentage ( ~ 5%) of immature erythrocytes—specifically polychromatic erythrocytes that lack nuclei required for the micronuclei assay—are prematurely released into the peripheral blood. When stained with acridine orange, polychromatic erythrocytes appear red due to the presence of cytoplasmic RNA, while micronuclei, as the only source of DNA for polychromatic erythrocytes, can be readily recognized by acridine orange and appear syellow-green.^183,184^

One major limitation of erythrocyte micronucleus assay accuracy is the spleen, which clears aged or damaged erythrocytes from circulation. For this reason, early erythrocyte micronucleus assay studies were often conducted in splenectomized animals. However, advancements in flow cytometry have significantly improved assay accuracy by reducing manual error, though splenic interference remains a factor to consider.^185–187^

In terms of application, erythrocyte micronucleus assay is particularly suitable for clinical testing rather than laboratory-based research. Its primary utility lies in assessing genotoxic damage already sustained by an individual, rather than evaluating cellular responses to experimentally applied genotoxic agents. This represents a key distinction between the erythrocyte micronucleus assay and cytokinesis-block micronucleus assay.

### Buccal micronucleus assay

In the 1980s, micronuclei have been discovered in buccal cells and have been used for more than four decades now.^183,188,189^ However, similar to erythrocyte micronucleus assay, it did not receive widespread attention when it first appeared. It was not until 2010 that its potential in genotoxicity evaluation was gradually recognized. Compared to the cytokinesis-block micronucleus assay and erythrocyte micronucleus assay, the primary advantage of the buccal micronuclei assay is its convenience and efficiency, as it does not require blood sampling, cell culture, or flow cytometry analysis. The protocols provide detailed steps for conducting buccal micronucleus assay.^190^ In brief, buccal cells are collected using a micro-brush, followed by gradient dilution to prepare a single-cell suspension. Cells are then centrifuged onto glass slides, fixed, stained, and examined under a fluorescence microscope.

In terms of application, buccal micronucleus assay is especially suitable for evaluating genetic damage induced by respiratory and oral irritants, such as smoking and alcohol consumption.^191,192^ For occupational exposure factors, buccal micronucleus assay is also commonly used for genotoxicity assessment of populations exposed to petrochemical pollution or dust workers.^193–195^ buccal micronucleus assay is gaining popularity for its simplicity and minimal invasiveness, showing higher sensitivity in low-dose radiation exposure and potentially assessing the genotoxicity of rare gases, suggesting that it may soon be integrated into routine toxicological protocols.^196,197^

### High-throughput micronucleus assay

High-throughput micronucleus assay refers to a class of emerging micronucleus assays characterized by the ability to rapidly and efficiently analyze target cell micronuclei and generate quantitative scores (essentially based on micronuclei staining). It originated from the application of flow cytometry in micronuclei detection in the 1990s.^198^ Initially, flow cytometry could not directly enumerate micronuclei; instead, it provided relative scores using staining markers, such as transferrin receptor (CD71).^198,199^ With advancements in both flow cytometry and imaging technologies, flow cytometry can now be combined with the cytokinesis-block micronucleus assay, targeting binuclear cells and automatically enabling imaging, thereby eliminating the manual and slow observation of traditional microscopy.^200,201^

However, despite its efficiency and accuracy, the high cost of flow cytometers currently limits their widespread adoption across research institutions. In fact, flow cytometer-independent high-throughput micronucleus assay have been developed, where in target cells are simply cultured in multi-well plates (e.g., 24/48/96-well plate), stained for DNA after external stimulation, and finally analyzed using automated imaging software. While this method does not surpass flow cytometry-based high-throughput micronucleus assay in speed or precision, it offers notable savings in time and reagent use compared to conventional microscopy-based approaches.^202,203^ At present, conventional micronucleus assays remain the most widely used and practical due to their simplicity and accessibility.

Despite the limitations—such as the inability to resolve micronuclei structure details, identify formation mechanisms, or distinguish single from multiple micronuclei—high-throughput micronucleus assay is emerging as a standard for large-scale micronuclei assays, pending further validation and refinement.^60,204^

### Micronuclei purification: the way forward

Micronuclei assays primarily rely on microscopic observation and counting, as micronuclei frequency serves as a reliable indicator of genotoxicity. However, investigating changes within micronuclei requires more than only counting. Consequently, purification of micronuclei for subsequent analyses, such as DNA sequencing and proteomic assays, has become a critical issue.

Selecting an appropriate method for micronuclei induction prior to purification is crucial. Low-dose radiation and specific chemotherapeutic agents, such as paclitaxel and cisplatin, can induce numerous micronuclei formation while maintaining a high cell density, which is essential for purification.^4^ These stimuli act as an “all-encompassing attack” on the entire genome, making them suitable for studying proteomic changes in micronuclei under specific genotoxic conditions. Targeting specific chromosomes may be more effective in inducing incorrect segregation and subsequent micronuclei formation to characterize chromosomal alterations within micronuclei. For instance, the Y chromosome, which lacks CENP-B, can be selectively inactivated by degrading CENP-A with minimal effects on other chromosomes (as CENP-B can substitute for CENP-A), generating Y chromosome-containing micronuclei in bulk. The Y chromosomes can be labeled with fluorescent probes to track changes within micronuclei, such as NHEJ and chromothripsis.^85,131,205^

Micronuclei purification using gradient sucrose centrifugation, a method initially proposed by Shimizu et al. in the 1990s, is widely accepted. This process involves inducing micronuclei in target cells using genotoxic agents (e.g., hydroxyurea and nocodazole), collecting the processed cells, and briefly incubating them in a serum-free medium containing cytochalasin B. The cells were then added to a Dounce pestle with an optimized lysate. The ground homogenate was placed on a gradient sucrose solution and centrifuged at low speed before collecting the micronuclei suspension, which was then placed in a 1.8 M sucrose solution for ultracentrifugation. Finally, a small amount of suspension is collected with a linear gradient of sucrose solution (1.1 M-1.8 M) to collect the upper part of the clear suspension, which contains the purified micronuclei.^4,98,206^

However, sucrose gradient centrifugation cannot differentiate between intact and ruptured micronuclei, necessitating distinct labeling for each type. One proposed approach takes advantage of the fact that cGAS can localize to ruptured micronuclei using a fluorescent tag (such as GFP) conjugated to cGAS and overexpressed in target cells. This method would facilitate the labeling and sorting of ruptured micronuclei by flow cytometry.^207^

Micronuclei purification combined with comprehensive proteomic analysis or genome sequencing is of significant importance and addresses several complex issues. For example, epigenetic reprogramming of the genome following cellular stimulation by genotoxic factors may result from micronuclei reincorporation. This process is crucial for understanding heritable genomic alterations in tumor cells in the face of radiotherapy.^7^

## Cancer and micronuclei

CIN is a hallmark of most cancers and plays a crucial role in enabling cancer cells to evolve and adapt to their environment. Micronuclei formation, a primary marker of CIN, is common in cancer cells, possibly due to their reduced sensitivity to DSBs.^208,209^ Generally, higher micronuclei frequency indicates greater chromosomal instability in cancer cells, correlating with increased malignancy.^210^ Historically, micronuclei were used as diagnostic indicators, such as Howell-Jolly bodies in acute leukemia. Recent research has revealed that micronuclei also serve as biomarkers, drive CIN, and contribute to tumor progression. The relationship between micronuclei and tumors has gained increasing prominence.^107,211^ Table 4 summarizes the frequency changes of micronuclei in various diseases, including several tumor types, as reported in studies from the past five years (Table 4).

**Table 4:** Overview of reports on micronucleus frequency of various diseases in the past five years

Disease	Method	Populations	Cell types	Cases (M ± SD/SE, N)	Controls (M ± SD/SE, N)	*p*	Reference
Bladder cancer	DAPI staining method	Spaniards	Bladder exfoliated cells	18.29 ± 10.04	14.40 ± 8.49	<0.01	^233^
Colon cancer	Cytokinesis-block micronucleus assay	Greeks	Lymphocytes	26.28 ± 6.30 (SD,27)	7.91 ± 1.14 (SD,10)	<0.001	^305^
Fanconi anemia	Buccal micronucleus assay	Spaniards and American	Buccal epithelium	4.0 ± 3.9 (SD,40)	1.3 ± 1.1 (SD,24)	<0.001	^306^
Chronic kidney disease	Cytokinesis-block micronucleus assay	Indians	Lymphocytes	6.69 ± 0.21 (SD,266)	1.40 ± 0.06 (SD,316)	<0.001	^307^
Heart Failure	Cytokinesis-block micronucleus assay	Serbs	Lymphocytes	23.31 ± 2.69 (SD,29)	8.95 ± 1.61 (SD19,)	<0.001	^308^
Endometrial cancer	Cytokinesis-block micronucleus assay	Serbs	Buccal epithelium	19.07 ± 2.89 (SD,30)	8.33 ± 1.83 (SD,30)	<.0005	^309^
Oral squamous cell carcinoma	Felgen staining method	NA	Buccal epithelium	21.64 ± 7.348 (SD,25)	0.44 ± 0.821 (SD,25)	<0.05	^310^
Sickle cell anemia	Cytokinesis-block micronucleus assay	Brazilians	Lymphocytes	2.17 ± 0.27 (SD,77)	0.23 ± 0.08 (SD,58)	*P* < 0.0001	^311^

### Cancer progression and micronuclei

Chronic activation of the cGAS-STING pathway is another mechanism by which micronuclei promote cancer progression, in addition to inducing genomic rearrangement.^108^ Typically, the cGAS-STING pathway is activated by cytoplasmic double-stranded DNA, triggering downstream reactions that promote IFN-I secretion to resist DNA damage stimuli. This activation mode usually hinders the progression of the early stages of cancer. However, tumors with high CIN can uniquely avoid the IFN-mediated killing effect and even use this pathway to promote their progression.^212^ For example, in some advanced tumors (e.g., skin melanoma and colon cancer), increased promoter methylation of cGAS and STING coding genes reduces their expression, silencing the cytoplasmic DNA sensing pathway, and evading immune surveillance.^213–215^ Additionally, HER2 in melanoma and colon cancer cells can recruit AKT1 to disrupt STING signaling and selectively inhibit TBK1-IRF3 signal transduction.^216,217^ Notably, direct silencing of cGAS and STING through methylation is not the primary mechanism by which tumors evade immune surveillance. In breast, lung, pancreatic, thyroid, and head and neck cancers, cGAS and STING promoter methylation is reduced, and mRNA levels are increased.^213,218^ In BT-549 cells (human triple-negative breast ductal carcinoma), high CIN cancer cells (constructed through MPS1 inhibitor-reverse) rely on the cGAS-STING-STAT3 and non-classical NF-κB pathways for survival, with IL-6 mediating the link between cGAS-STING and STAT3.^21^ In uveal melanoma, progression is often driven by the loss of polynuclear inhibitory complex 1 (PRC1), leading to chromosomal segregation errors and micronuclei formation. This abnormally activates the cGAS-STING-mediated tumor cell inflammatory response, significantly reducing patient survival and prognosis.^217^ Furthermore, when tumor cells lack P53 or P21, chronic activation of the cGAS-STING pathway stimulates senescence-associated secretory phenotype (SASP), establishing an immunosuppressive tumor microenvironment that promotes tumor progression.^219,220^

Notably, cGAS can function independently in the nucleus, although its nuclear entry mechanism is not fully understood. It has been proposed that during interphase, nuclear cGAS may result from contact between the nuclear envelope of the previous mitosis and nuclear DNA during cleavage.^24,221^ However, nuclear localization of cGAS may occur even with an intact nuclear envelope, particularly in the presence of DNA damage.^222^ Once in the nucleus, cGAS can promote tumor development by inhibiting homologous recombination repair, exacerbating DNA damage, although its response to nuclear DNA is weaker than to cytoplasmic DNA.^95,223^

Micronuclei also contribute to tumor heterogeneity. For example, BAP1-inactivated melanoma exhibits strong heterogeneity in cell morphology, including nuclear budding, micronuclei, and shaded nuclei.^224^ In primary liver cancer characterized by intratumoral genetic heterogeneity, micronuclei and chromosomal breaks contribute to IGH formation.^225^ Abnormal nuclear morphology, typically observed in polyploid giant cancer cells (PGCC), is associated with poor tumor prognosis. PGCCs bypass specific mitotic stages to form nuclei with various shapes, including micronuclei, increasing nuclear heterogeneity, and promoting cancer cell genome evolution.^226–228^

The role of micronuclei in tumor progression is complex and often indirect. Although not directly related to tumor progression, the downstream pathways activated by micronuclei or the cellular activities they induce are closely associated with cancer progression.

### Cancer prediction and micronuclei

Micronuclei frequency offers a significant advantage in cancer risk prediction because it is present in essentially all cancer types. Although micronuclei are less sensitive in predicting certain cancer risks (e.g., lung cancer) compared to nucleoplasmic bridges (NPBs) and nuclear buds (NBUDs), they are the most consistent indicators of genomic instability. In the original prediction model, spontaneous micronuclei, NPBs, and NBUDs were associated with 2-fold, 29-fold, and 6-fold increases in cancer risk, respectively.^229^ However, the potential of micronuclei frequency as a cancer biomarker remains undeniable, despite the limitations of NPBs, which can be lost due to “bridge-breaking,” and NBUDs, which can vary due to fluctuations in folate levels in the body.^230^ In prostate cancer, peripheral blood lymphocytes of patients did not show significantly higher micronuclei frequency compared to the control group. However, the spontaneous frequency of NPBs and NBUDs increases considerably.^231,232^ Conversely, micronuclei demonstrated excellent predictive ability in bladder cancer. The frequency of micronuclei in urethral exfoliated cells of bladder cancer patients is higher than that of healthy individuals, and patients with recurrent bladder cancer show significantly higher frequencies than those with first-episode bladder cancer.^233^ Moreover, micronuclei assays in peripheral blood lymphocytes can also predict bladder cancer risk.^234^

In colorectal cancer (CRC), circulating tumor-associated macrophages are commonly used for prediction, unlike in lung cancer, which often utilizes peripheral blood lymphocytes. Importantly, micronuclei in CRC are not associated with genotoxic therapy (such as chemotherapy) or tumor staging but are only associated with tumor response. However, their predictive role in progression-free survival (PFS) and overall survival in CRC patients serves as an independent prognostic indicator.^235^ Importantly, micronuclei frequency can distinguish between progressive and stable CRC with significant specificity.^236^

In addition to cancer risk and recurrence prediction, micronuclei frequency has been associated with precancerous lesions and treatment-related complications. For instance, in chronic obstructive pulmonary disease (COPD) and lung cancer, micronuclei formation causes chromosomal breaks that lead to tissue remodeling and malignant transformation in susceptible smokers.^237^ Additionally, micronuclei frequency reflects side effects after radiotherapy for cervical cancer, such as burns, erythema, and fistulae.^238^

In conclusion, micronuclei demonstrate a strong association with cancer and show great potential as a diagnostic tool. However, further reliable clinical studies are needed to validate their efficacy in cancer screening.

### Cancer treatments and micronuclei

Micronuclei, while typically associated with CIN, may possess anti-tumor potential as suggested by recent studies.^239,240^ Unlike genotoxic drugs, DNA polymerase Q (POLQ) inhibitors can induce micronuclei formation by blocking the microhomology-mediated end joining (MMEJ) repair pathway.^241^ In pancreatic ductal adenocarcinoma (PDAC), approximately 20% of patients have mutations in HR genes (such as BRCA1, BRCA2, and ATM), and POLQ knockout stimulates micronuclei formation and promotes IFN secretion. This leads to CD8 + T cell activation and infiltration in BRCA2-mutated PDAC, impeding tumor progression.^242^

Inhibiting DNA repair to generate micronuclei and activate immune pathways represents only a preliminary aspect of the current use of micronuclei in anti-tumor therapy. Thymidine kinase-related protein kinase (TTK), also referred to as monopolar spine 1 kinase (MPS1), is a fundamental regulatory component of the spindle assembly checkpoint and is primarily involved in its activation to ensure the accurate segregation of chromosomes into daughter cells. Consequently, TTK inhibitors induce micronuclei production by disrupting mitosis. TTK inhibitors activate the cGAS-STING pathway and induce SASP secretion in breast and liver cancer cells. High concentrations of SASP recruit various immune cells to eliminate tumors.^243,244^ Currently, the TTK inhibitor CFI-402257 has been approved by the US Food and Drug Administration (FDA) for clinical trials.

When the induction of micronuclei formation is considered a potential cancer treatment strategy, certain proteins that were previously highly expressed in tumors and deemed adverse factors may also become targets for exploitation. The mouse double minute 2 (MDM2) protein is an E3 ubiquitin-protein ligase capable of degrading p53 through ubiquitination.^245^ Therefore, the development of MDM2 inhibitors appears to be promising. MDM2 sustains the progression of DNA replication forks, antagonizes PARP1, and promotes micronuclei formation. Although it induces significant CIN, it also results in the extensive death of camptothecin-sensitive cancer cells.^246^ Furthermore, in radiation therapy, the effect of radiation sensitization can be achieved by inducing micronuclei formation. AZD6738 is an oral ATR inhibitor that can attenuate radiation-induced G2 cell cycle checkpoints and inhibit HR from forming micronuclei in vivo, thereby resulting in radiation sensitization.^247,248^

Inducing micronuclei formation appears to attenuate drug resistance in tumor cells to some extent. A notable feature of the KRAS-LKB1 (KL) mutant is STING silencing, which involves two pathways: mitochondrial dysfunction and reduced accumulation of 2′3′- cGAMP, resulting in T-cell rejection and resistance to PD-1 therapy. However, transient MPS1i can rapidly generate a large number of micronuclei, activate STING, restore T cell infiltration, and enhance the efficacy of PD-1.^249,250^ Paclitaxel, a first-line anti-cancer drug, although demonstrates promising therapeutic effects in patients with various malignant tumors, inevitably encounters resistance. The mechanism by which paclitaxel exerts its cytotoxic effects is similar to that of genotoxic stimuli, leading to micronuclei production. It disrupts microtubule function, causes cell cycle arrest, induces micronuclei formation, and increases the CIN of cancer cells, thereby resulting in cancer cell death.^251^ Consequently, theoretically, any factor that inhibits the formation of cancer cell micronuclei can contribute to paclitaxel resistance. For instance, in ovarian cancer, the absence of lamin A/C generally results in an increased sensitivity to paclitaxel. However, exogenous overexpression of lamin A/C significantly increases the resistance of ovarian cancer cells to paclitaxel.^252^

Theoretically, activating the cGAS-STING pathway by inducing micronuclei to exert anti-tumor effects may be a viable strategy. However, from the current perspective, this is an undeniably risky approach. The effects of micronuclei formation are not easily controllable, and there is a considerable likelihood that it may promote cancer progression. Although recent studies have demonstrated the potential to activate STING by encapsulating its own DNA in exosomes and subsequently releasing it into host immune cells, the use of micronuclei for anti-tumor effects still necessitates more evidence.^253,254^

In conclusion, a recent review by AI-Rawi et al. is noteworthy, as it addresses the broader topic of CIN and offers new insights into cancer treatment.^255^

## Other diseases and micronuclei

Although the link between micronuclei and cancer has been extensively studied, their roles in other diseases have garnered increasing attention in recent years. Micronuclei are increasingly recognized as valuable biomarkers in various conditions, including neurodegenerative disorders, reproductive diseases, and genetic disorders such as Fanconi anemia (FA). Despite the limited research on micronuclei in these conditions, accumulating evidence suggests that they may play a crucial role in their pathophysiology.

### Neurodegenerative diseases and micronuclei

Neurodegenerative diseases are characterized by the progressive deterioration of neuronal function and structure, leading to cognitive, motor, and other functional impairments. Evidence suggests that patients with Alzheimer’s disease (AD) exhibit an elevated frequency of micronuclei in peripheral blood lymphocytes, potentially associated with chromosome missegregation. These micronuclei often contain intact chromosomes.^256^ A plausible explanation for this phenomenon is the dysregulation of O-GlcNAc modification mediated by O-GlcNAc transferase (OGT) and O-GlcNAcase (OGA). O-GlcNAcylation plays a crucial role in AD progression by influencing Tau protein phosphorylation and aggregation. During the cell cycle, O-GlcNAcylation levels peak in the M-phase, coinciding with increased OGT levels and requiring regulation by OGA degradation. The absence of OGA results in abnormal chromosome segregation and impaired cytoplasmic division, leading to prolonged M-phase. Cells that are unable to complete division exhibit genomic instability and cell death, while surviving cells commonly form micronuclei due to abnormal chromosome segregation.^257–259^ Amyloid beta-42 accumulation, a hallmark of AD, may induce DNA damage, particularly under folate-deficient conditions, although no definitive link between amyloid protein and folate has been established.^260^ Patients with AD tend to have shorter telomeres in nerve cells, a phenomenon also observed in AD mice (APP/PS1). The accelerated telomere shortening in APP/PS1 mouse brains and age-related increase in micronuclei are consistent with the presence of complete chromosomes in the micronuclei of Alzheimer’s disease patients.^261^ However, a recent study found no significant difference in micronuclei frequency in buccal mucosal cells between AD patients and healthy individuals, contradicting earlier findings.^262^

Parkinson’s disease (PD), another prevalent neurodegenerative disorder, is characterized by chromosomal breaks in peripheral blood lymphocytes, with scattered chromosomal fragments forming micronuclei. FISH analysis reveals a higher percentage of centromere-negative micronuclei, supporting the theory that these micronuclei originate from chromosomal fragmentation in PD.^256^ Interestingly, despite increased oxidative DNA damage markers (such as 8-oxo-7,8-dihydro-2’-deoxyguanosine) in peripheral blood lymphocytes, PD patients treated with levodopa did not show increased micronuclei frequency.^263^

However, research on micronuclei in other neurodegenerative diseases is limited. In Huntington’s disease, caused by an N-terminal polyglutamine amplification mutation in the Huntington protein (mHTT), embryonic stem cells from patients show significantly increased micronuclei.^264^ Multiple sclerosis patients exhibit increased micronuclei frequency in buccal mucosal cells, with a decrease observed following gamma ray therapy.^265^

Although these findings suggest a potential role for micronuclei in neurodegenerative diseases, it remains challenging to determine the downstream pathways activated by micronuclei that may promote disease development. Further research is required to elucidate the significance of micronuclei in the pathogenesis of neurodegenerative disorders.

### Reproductive disorders and micronucleus

Humans have evolved mechanisms to protect their reproductive cells more effectively than other cell types against external factors because damage to reproductive cells is significantly more detrimental to the human body. The presence of micronuclei in sperm has been identified as a risk indicator for infertility and is positively correlated with DNA damage.^266^ These micronuclei are associated with abortive apoptosis, an incomplete or prematurely halted apoptotic process that can induce chromothripsis. Research on abortive apoptosis has primarily focused on male germ cells. The discovery of apoptotic structures and overexpression of fatty acid synthase (FAS) receptors in infertile male sperm prompted researchers to propose the hypothesis of abortive apoptosis in male germ cells.^145,267^ When cells are exposed to severely harmful stimuli (such as radiation or hypoxia), the apoptotic process is activated. Although most cells die, a small subset may survive with minor damage. These cells must repair damaged genetic material, a process prone to errors that can lead to chromothripsis.^268^

Infertility can result from external factors and abnormal cellular activity in embryonic cells. External physical or chemical factors may increase micronuclei formation in patients with infertility. In embryonic cells, arrest of DNA replication forks during the S phase can persist in the G2 phase. During cell division, incomplete DNA replication relies on ATR and MRE11 to form DNA damage foci, similar to DSBs, leading to spontaneous chromosome breakage and micronuclei formation. This process results in frequent aneuploidy in embryonic cells, negatively affecting embryo quality.^269^ The unification of paternal and maternal genomes after fertilization is a highly error-prone process. Parental genomes polarize towards each other, driven by centrosomes, microtubules, and nuclear pore complexes, ultimately aggregating in the pronucleus. Although inefficient, this process is essential for the formation of healthy diploid embryos. However, clustering often fails, leading to chromosomal missegregation and micronuclei formation, which disrupts embryonic development.^270^

Despite their vulnerability to external factors and internal cellular activity, embryos attempt to maintain genomic stability. In some primate blastomeres, extensively damaged micronuclei are sealed, preventing them from entering the blastocyst. These damaged blastomeres are fragmented and eliminated, thus mitigating the impact of aneuploidy on embryonic development.^118^

### Fanconi anemia and micronucleus

FA is a rare genetic disorder characterized by bone marrow failure, developmental abnormalities, congenital malformations, and increased susceptibility to cancer. Multiple genes associated with FA form the FA pathway and are involved in DNA repair processes. At the cellular level, FA is primarily characterized by an extremely unstable genome and a high prevalence of micronuclei.^271,272^

Abnormalities in the FA/BRCA pathway underlie impaired DNA repair in patients with FA. The first step in this pathway is DNA damage recognition by the FA core complex (comprising FANCA, FANCB, FANCC, FANCE, FANCF, FANCG/XRCG9, FANCL, and FANCM), which identifies and localizes DNA damage. FANCM can unwind DNA crosslinks.^273^ Subsequently, FANCL catalyzes the ubiquitination of FANCI-FANCD2, which is recruited to the damage site, a critical step in the FA/BRCA pathway.^272^ The FANCI-FANCD2 complex activates nucleic acid endonucleases, such as SLX4 and FAN1, near the DNA damage site, releasing DNA cross-links and producing single-stranded DNA (ssDNA). HR then occurs with the assistance of DNA polymerases, such as polθ, BRCA1, and BRCA2, to complete DNA repair.^274–278^ Mutations in any FA pathway gene can lead to pathway failure, blocked DNA repair, chromosomal breaks, and micronuclei formation. The absence of Fancd2 appears to result in a more destabilized genome, as evidenced by increased chromosome breaks and micronuclei formation in mouse cells.^279^

Given the high prevalence of micronuclei in patients with FA, the frequency of micronuclei is potentially an effective diagnostic indicator for FA. A study suggested that low-dose radiation irradiation of mitomycin (MMC)-treated cells from FA patients induces the formation of numerous micronuclei, providing a simple and rapid diagnostic method for FA.^280^

## Conclusion and Perspective

The past decade has witnessed significant advancements in micronuclei research, expanding our understanding of their role beyond that of a toxicological indicator. However, numerous uncertainties remain.

The 2017 identification of the cGAS-STING pathway associated with micronuclei marked the beginning of a new era, with gradual elucidation of this pathway and its mediated immune response.^24^ Recent studies have challenged the foundational theory of this pathway. Nucleosomes in genotoxic damage-induced micronuclei impede the binding of micronuclear DNA to cGAS, thereby limiting downstream immune signal transmissions. The activation of cGAS by micronuclei appears to depend on its pre-ruptured chromatin state.^98–100^ These findings suggest that inducing micronuclei to trigger the cGAS-STING pathway immune response may be ineffective for tumor treatment. Moreover, if tumor immunity through this pathway cannot be harnessed within acceptable limits, it may lead to CIN due to excessive micronuclei production, potentially harming the patients.

The precise content of micronuclei is still unclear. Proteomic analyses of micronuclei induced by various genotoxic factors have revealed that their protein composition differs from that of the primary nucleus and varies depending on the inducing factor.^4^ This raises questions about whether micronuclei inclusion is factor-dependent rather than cell-dependent. Cellular epigenetic reprogramming induced by micronuclei formation is gaining attention. Purifying micronuclei and an assay for transposition accessible chromatin with high-throughput sequencing (ATAC-seq) have revealed the predominance of heterochromatin inside micronuclei, suggesting potential transcriptional regulation (although only low transcriptional activity has been confirmed). Currently, micronuclei appear to be random products with variable effects on human health. Even chromothripsis occurring within micronuclei has been proven to cure congenital diseases.^163^

Future research challenges include determining the overall impact of micronuclei, identifying diseases associated with intact and ruptured micronuclei, understanding the directionality of micronuclei formation, and targeting beneficial micronuclei. Moreover, most current research on micronuclei focuses on statistical frequency data and in vitro experiments, with limited in vivo studies. This highlights the need for more comprehensive research to deepen our understanding of micronuclei and their biological implications. Finally, a standardized and widely applicable micronuclei assay is urgently needed. Although buccal cell micronuclei assay has shown potential as an essential indicator for evaluating human gene stability, more clinical evidence is required for its widespread adoption.
